# Isolation, Structural Characterization and Macrophage Activation Activity of an Acidic Polysaccharide from Raspberry Pulp

**DOI:** 10.3390/molecules27051674

**Published:** 2022-03-03

**Authors:** Yongjing Yang, Xingxing Yin, Dejun Zhang, Jie Lu, Xuehong Wang

**Affiliations:** 1 College of Ecological and Environmental Engineering, Qinghai University, Xining 810016, China; y200713000515@qhu.edu.cn (X.Y.); 1998990035@qhu.edu.cn (D.Z.); ys210854000307@qhu.edu.cn (J.L.); ys210713000141@qhu.edu.cn (X.W.); 2 State Key Laboratory of Plateau Ecology and Agriculture, Qinghai University, Xining 810016, China

**Keywords:** raspberry pulp, acidic polysaccharide, structural characterization, macrophage activation

## Abstract

The discovery of safe and effective plant polysaccharides with immunomodulatory effects has become a research hotspot. Raspberry is an essential commercial fruit and is widely distributed, cultivated, and consumed worldwide. In the present study, a homogeneous acidic polysaccharide (RPP-2a), with a weight-average molecular weight (Mw) of 55582 Da, was isolated from the pulp of raspberries through DEAE-Sepharose Fast Flow and Sephadex G-200 chromatography. RPP-2a consisted of rhamnose, arabinose, galactose, glucose, xylose, galacturonic acid and glucuronic acid, with a molar ratio of 15.4:9.6:7.6:3.2:9.1:54.3:0.8. The results of Fourier transform infrared spectroscopy (FT-IR), gas chromatography-mass spectrometer (GC-MS), 1D-, and 2D-nuclear magnetic resonance (NMR) analyses suggested that the backbone of RPP-2a was primarily composed of →2)-*α*-L-Rha*p*-(1→, →2,4)-*α*-L-Rha*p*-(1→, →4)-*α*-D-GalA*p*-(1→, and →3,4)-*α*-D-Glc*p*-(1→ sugar moieties, with side chains of *α*-L-Ara*f*-(1→, *α*-L-Ara*p*-(1→, and *β*-D-Gal*p*-(1→3)-*β*-D-Gal*p*-(1→ residues linked to the O-4 band of rhamnose and O-3 band of glucose residues. Furthermore, RPP-2a exhibited significant macrophage activation activity by increasing the production of nitric oxide (NO), tumor necrosis factor-α (TNF-α), interleukin-6 (IL-6), interleukin-1β (IL-1β), and the expression of inducible nitric oxide synthase (iNOS) and cytokines at the transcriptional level in RAW264.7 cells. Overall, the results indicate that RPP-2a can be utilized as a potential natural immune-enhancing agent.

## 1. Introduction

Polysaccharides, naturally occurring flexible macromolecular polymers with complex structures, are the primary components of plants, fungi, bacteria, algae, and even animals [1]. Polysaccharides possess numerous pharmacological activities, including anti-tumor, anti-inflammatory, antioxidant, antimicrobial, antidiabetic, immunomodulatory, etc. [2,3,4]. Their immunoregulatory effects are considered to be the primary activity of polysaccharides. Hence, several polysaccharides are widely utilized as potent immunomodulators in the food and medicine industries [5]. Previous studies have reported that most polysaccharides-induced actions are dependent on macrophage functional ability [6]. Macrophages are the first line of defense, with various activities performed by the multitudinous immune cells, such as phagocytosis, surveillance, chemotaxis, and destruction of targeted organisms [7,8,9]. Research has been proven that macrophage activation is a promising approach to improve host immune capability and strengthen disease resistance [10]. Accumulating evidence suggests that plant-derived polysaccharides, with relatively low toxicity and side effects, possess potent immunomodulatory activity by enhancing or activating the immune responses of macrophages [8,11]. For instance, a polysaccharide from the wall of *Sambucus adnate* exerts an immunomodulatory effect by activating macrophages and enhancing the host’s immune system function [12]. Similarly, the polysaccharides obtained from Radix aconiti significantly promote macrophage phagocytosis and increase the secretion of biological factors [13]. Hence, attempting to discover a safe and effective plant polysaccharide with a potent immunomodulatory effect has become a hot spot in research that is gaining increasing attention worldwide.

Raspberry (*Rubus idaeus* L.), a perennial shrub belonging to the diverse *Rubus genus* rank, is an essential commercial fruit that is widely distributed, cultivated, and consumed worldwide [14]. It is usually consumed as fresh fruit or processed into jams, juices, and wines, or served as an ingredient in other products and various foods [15]. In recent years, the raspberry has emerged as the most popular berry due to the presence of numerous bioactive substances, including flavonoids, tannins, phenolic acids, stilbenoids, polysaccharides, vitamins, and minerals [16]. Moreover, the dietary intake of raspberries has been used to treat cardiovascular diseases, obesity, cancer, and degenerative diseases [17]. Polysaccharides are one of the most important bioactive components in raspberries. So far, many bioactive polysaccharides have been isolated from the raspberry. For instance, Yu et al. [18] isolated and purified a heteropolysaccharide from the raspberry fruit, which exhibited excellent antioxidant and antiglycation activities. Xu et al. [19] purified a heteropolysaccharide (RCPI) and obtained a degraded polysaccharide (DRCPI) from the raspberry fruits that displayed high antioxidant activity and thermal stability. Similarly, Ke et al. reported that the raspberry polysaccharides could resist palmitic acid-induced lipotoxicity and ethylcarbamate-induced toxicity [16,20]. Our previous study found that crude raspberry pulp polysaccharides (RPPs) exhibited significant antitumor activity and chemotherapy enhancement effects in vivo by enhancing the cellular immune response of the host organism without any lesions in the liver or kidney tissues [21]. These results indicated that the polysaccharides derived from raspberries could be used as healthcare foods, dietary supplements, or medicines. In this study, an acidic polysaccharide from the pulp of raspberries was isolated and its structure and macrophage activation activity were investigated. This study aims to provide an effective plant polysaccharide for potential application as a natural immune-enhancing agent in functional food supplements or drugs.

## 2. Results

### 2.1. Isolation and Purification of Homogeneous Acidic Polysaccharide (RPP-2a)

**Figure 1:**
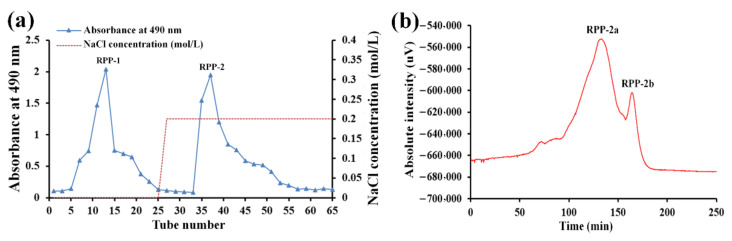
(**a**) Elution profiles of RPPs by DEAE-Sepharose Fast Flow chromatography and (**b**) RPP-2 on a Sephadex G-200 column.

The crude RPPs were purified by DEAE-Sepharose Fast Flow chromatography and a Sephadex G-200 column. As depicted in Figure 1a, two fractions designated RPP-1 and RPP-2 were eluted with 0 M and 0.2 M NaCl solutions and collected from the DEAE-Sepharose Fast Flow chromatography system. In our previous study, the properties of RPP-1 were extensively studied. The present study focused on RPP-2 only. RPP-2 was further purified by a Sephadex G-200 column. As illustrated in Figure 1b, two peaks designated RPP-2a and RPP-2b were observed. Compared to RPP-2b, RPP-2a exhibited a larger peak area, suggesting that it was the major ingredient in RPP-2. Thus, RPP-2a was concentrated, dialyzed, and lyophilized for further analysis.

### 2.2. The Monosaccharide Composition of RPP-2a

**Figure 2:**
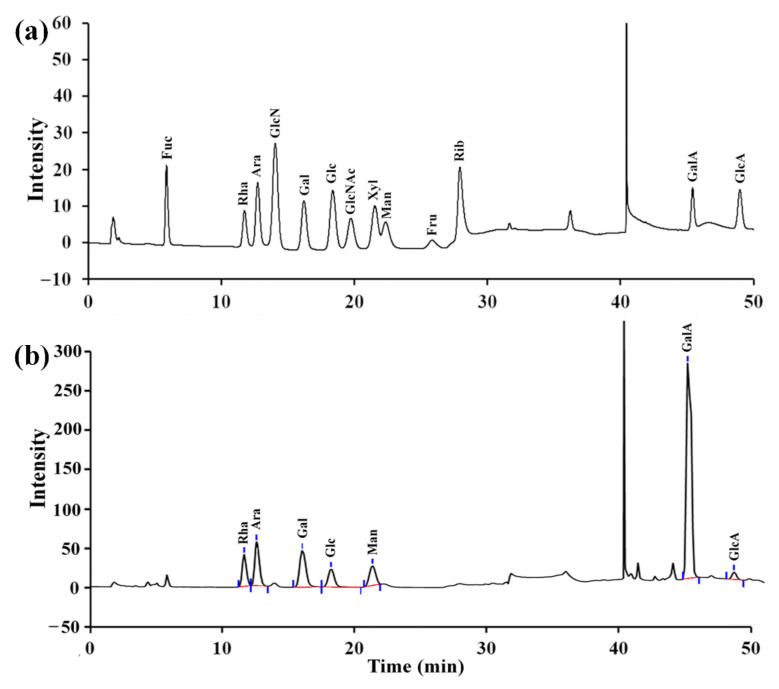
Monosaccharide composition of RPP-2a. (**a**) The profile of mixed monosaccharides standards and (**b**) monosaccharides in RPP-2a.

As depicted in Figure 2a, RPP-2a was composed of rhamnose (Rha), arabinose (Ara), galactose (Gal), glucose (Glc), xylose (Xyl), galacturonic acid (GalA), and glucuronic acid (GlcA), with a molar ratio of 15.4:9.6:7.6:3.2:9.1:54.3:0.8. Among the components, the galacturonic acid content was the highest.

### 2.3. Fourier Transform Infrared Spectrophotometer (FT-IR) Spectrum of RPP-2a

**Figure 3:**
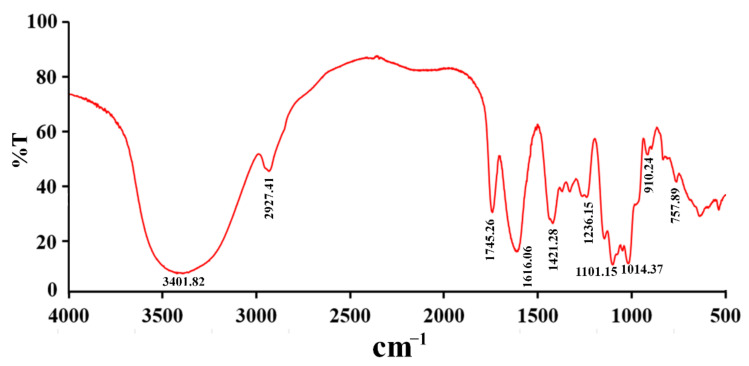
FT-IR spectra of RPP-2a ranging from 400 to 4000 cm^−1^.

The FT-IR spectrum of RPP-2a is illustrated in Figure 3. RPP-2a displayed the characteristic absorption peaks of polysaccharides at 3401.82 cm^−1^, 2927.41 cm^−1^, and 1421.28 cm^−1^ [22]. The band that appeared at 3401.82 cm^−1^ corresponded to -OH stretching vibration [23,24]. The absorption peaks at 2927.41 cm^−1^ and 1421.28 cm^−1^ were related to the stretching vibration of CH [25]. The intense peaks at 1745 and 1616 cm^−1^ corresponded to the symmetric and asymmetric C=O stretching vibration [26], and the absorption bands observed at 910 and 757 cm^−1^ suggested that RPP-2a was comprised of a pyranose structure [27]. The absorbance peaks at 1200–1000 cm^−1^ were assigned to the contribution of C-OH or C-O-C stretching vibration [28].

### 2.4. The Molecular Weight of RPP-2a

**Figure 4:**
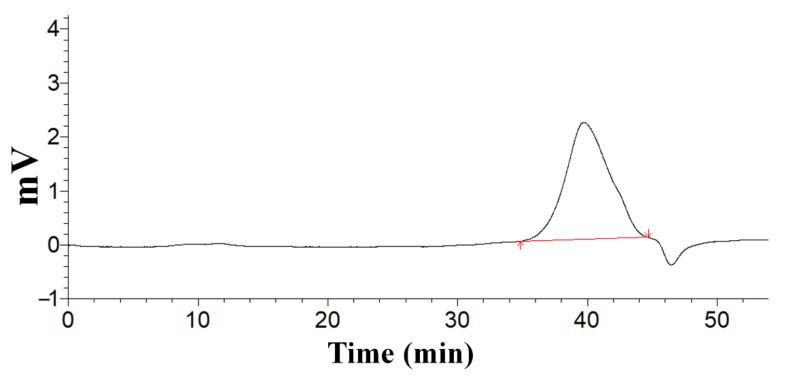
The homogeneity and molecular weight of RPP-2a.

A single and symmetrical peak was obtained for RPP-2a from the HPGPC, suggesting that the purified polysaccharide was homogeneous (Figure 4). The weight-average molecular weight (Mw), number-average molecular weight (Mn), and peak molecular weight (Mp) of RPP-2a were 55582 Da, 38824 Da, and 45471 Da, respectively. Mw/Mn is a measure of the width of the molecular weight distribution [29]. The Mw/Mn value of RPP-2a was 1.432.

### 2.5. Methylation and Gas Chromatography-Mass Spectrometry (GC-MS) Analysis of RPP-2a

**Figure 5:**
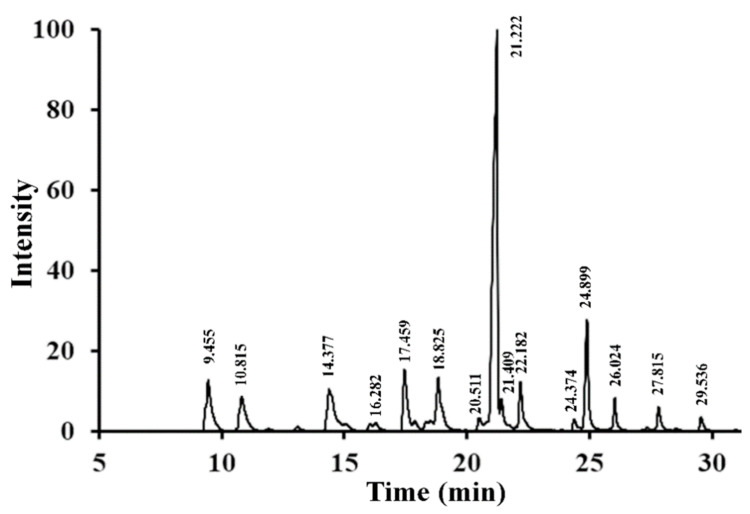
The GC-MS chromatogram of PMAAs of RPP-2a.

As depicted in Figure 5, 15 major peaks were present in the GC-MS spectrum of RPP-2a. The peaks were assigned based on the Complex Carbohydrate Structure Database developed by the Complex Carbohydrate Research Center of the University of Georgia (https://ccrc.uga.edu/ accessed on 2 January 2022). The results, including methylated sugars, mass fragment information, glycosyl linkage patterns, and molar ratios, are listed in Table 1. RPP-2a was composed of 15 types of glycosidic linkages, of which the percentages of →2)-Rha*p*-(1→, →2,4)-Rha*p*-(1→, →4)-Gal*p*-(1→, and →3,4)-Glc*p*-(1→ were 7.11%, 5.53%, 44.35, and 9.81%, respectively. These results indicated that RPP-2a was a branched heteropolysaccharide, and →2)-Rha*p*-(1→, →4)-Gal*p*-(1→ and/or →3,4)-Glc*p*-(1→ were the primary glycosidic linkages in the backbone. The non-reducing terminals were mainly composed of Ara*f*-(1→, Ara*p*-(1→, and Gal*p*-(1→ with the percentages of 7.61%, 5.69%, and 6.22%, respectively.

### 2.6. Nuclear Magnetic Resonance (NMR) Spectra of RPP-2a

For further structural analysis, 1D- and 2D-NMR analyses were performed. Combined with the methylation analysis and the 2D-NMR spectra, the ^1^H and ^13^C signals of these glycosidic linkages in RPP-2a were assigned and listed in Table 2. Although significant signal overlap and broad signals due to high molecular weight precluded definitive sequence determination, several characteristic peaks of the monosaccharide residues could be assigned. A total of 12 glycosyl residues were found in RPP-2a and designated as residues A, B, C, D, E, F, G, H, I, J, K, and L for convenience. Residue A was identified as →2)-*α*-L-Rha*p*-(1→ by its anomeric signals at δ_H_/δ_C_ 5.22/101.1 [2,30]. The anomeric signals at δ_H_/δ_C_ 5.21/93.78 and 4.5/97.63 corresponded to the residues →2)-*α*-L-Rha*p* (B) and →2)-*β*-L-Rha*p* (L), respectively [31]. Residues C and G were identified as →2,4)-*α*-L-Rha*p*-(1→ and *α*-L-Ara*p*-(1→ by the anomeric signals at δ_H_/δ_C_ 5.17/99.5 and 4.98/109.23, respectively [32]. The residue *α*-L-Ara*f*-(1→ (D) was matched with the signal of δ_H_/δ_C_ 5.08/108.45 [7]. Residue E was found to have a downfield H-1 (5.04 ppm) and a downfield C-1 (98.7 ppm), consistent with →3,4)-*α*-D-Glc*p*-(1→ [33]. Residue K had an anomeric proton signal and a carbon signal at 4.52 ppm and 104.69 ppm, which were assigned to →3)-*β*-D-Gal*p*-(1→ [34]. The anomeric signals at δ_H_/δ_C_ 4.99/100.39 and 4.56/105.74 were attributed to →4)-*α*-D-GalA*p*-(1→ (residue F) and *β*-D-Gal*p*-(1→ (residue J), respectively [35,36]. The anomeric signals at δ_H_/δ_C_ 4.56/105.74 and 4.85/101.88 were assigned to the methyl-esterified →4)-*α*-D-GalA*p*-(1→ (I) and →4)-*α*-D-GalA*p*-(1→(H) residues [37,38]. In general, the anomeric signals above the 4.8 ppm region represent *α*-configuration and the signals less than 4.8 ppm represent *β*-configuration [39,40]. Thus, the anomeric proton signals at δ_H_ 5.22, 5.21, 5.17, 5.08, 5.04, 4.99, 4.98, 4.85, and 4.84 ppm suggested the presence of *α*-configuration pyranose units [41], while the anomeric proton signals at 4.56, 4.52, and 4.5 ppm implied the presence of *β*-configuration pyranose units [42,43]. Additionally, the signal at δ_C_ 172.19 was assigned to the carboxylic carbon of *α*-Gal*p*A and the signal at δ_H_ 3.74/δ_C_ 54.5 was assigned to the methyl group of *α*-Gal*p*A on O-2/3 [36]. In the DEPT-135 spectra, the signal at δ 62.64 was assigned to the C-5 of D and G, the signal at δ 62.11 was assigned to the C-6 of J, and the signal at δ 62.26 was assigned to the C-6 of E and K.

The HMBC spectrum was further analyzed to determine the sugar sequences and linkage positions of RPP-2a. In the HMBC map, a correlation peak was observed between the C2 (δ 77.86) of A and H1 (δ 5.17) of C, implying the presence of the glycosidic bond →2,4)-*α*-L-Rha*p*-(1→2)-*α*-L-Rha*p*-(1→ [44,45]. The cross peak between the H4 (δ 4.36) of I and C1 (δ 101.1) of A indicated the presence of →2)-*α*-L-Rha*p*-(1→4)-*α*-D-GalA*p*-(1→ fragment. The C4 (δ 109.16) of H associated with the H1 (δ 4.84) of I implied the presence of →4)-*α*-D-GalA*p*-(1→4)-*α*-D-GalA*p*-(1→ linkage [38,46]. Besides, the connection manner of →4)-*α*-D-GalA*p*-(1→4)-*α*-D-GalA*p*-(1→ was speculated to exist through the HMBC correlation from C1 (δ 101.88) of H to H4 (δ 4.33) of F and C4 (δ 79.68) of F to H1 (δ 4.85) of H [47,48]. The correlation from C1 (δ 100.39) of F to H4 (δ 4.27) of E implied the presence of 4)-*α*-D-GalA*p*-(1→3,4)-*α*-D-Glc*p*-(1→ linkage [49]. The cross peaks between H1 (δ 5.08) of D and C4 (δ 78.18) of C, and H1 (δ 4.98) of G and C3 (δ 83.3) of E were observed in the HMBC spectrum, implying the presence of side chains of *α*-L-Ara*f*-(1→2,4)-*α*-L-Rha*p*-(1→ and *α*-L-Ara*p*-(1→3,4)-α-D-Glc*p*-(1→, respectively [32,50]. Moreover, in the NOESY spectrum, the correlation signal at δ 4.56/3.6 ppm was assigned to H1/H3 between the residues J/K, indicating that the →3)-*β*-D-Gal*p*-(1→ residue was terminated by *β*-D-Gal*p*-(1→ residue, and the cross peak between H1 (δ 4.52) of K and H3 (δ 4.01) of E indicated a connection manner of →3)-*β*-D-Gal*p*-(1→3,4)-*α*-D-Glc*p*-(1→ [44,51].

**Figure 6:**
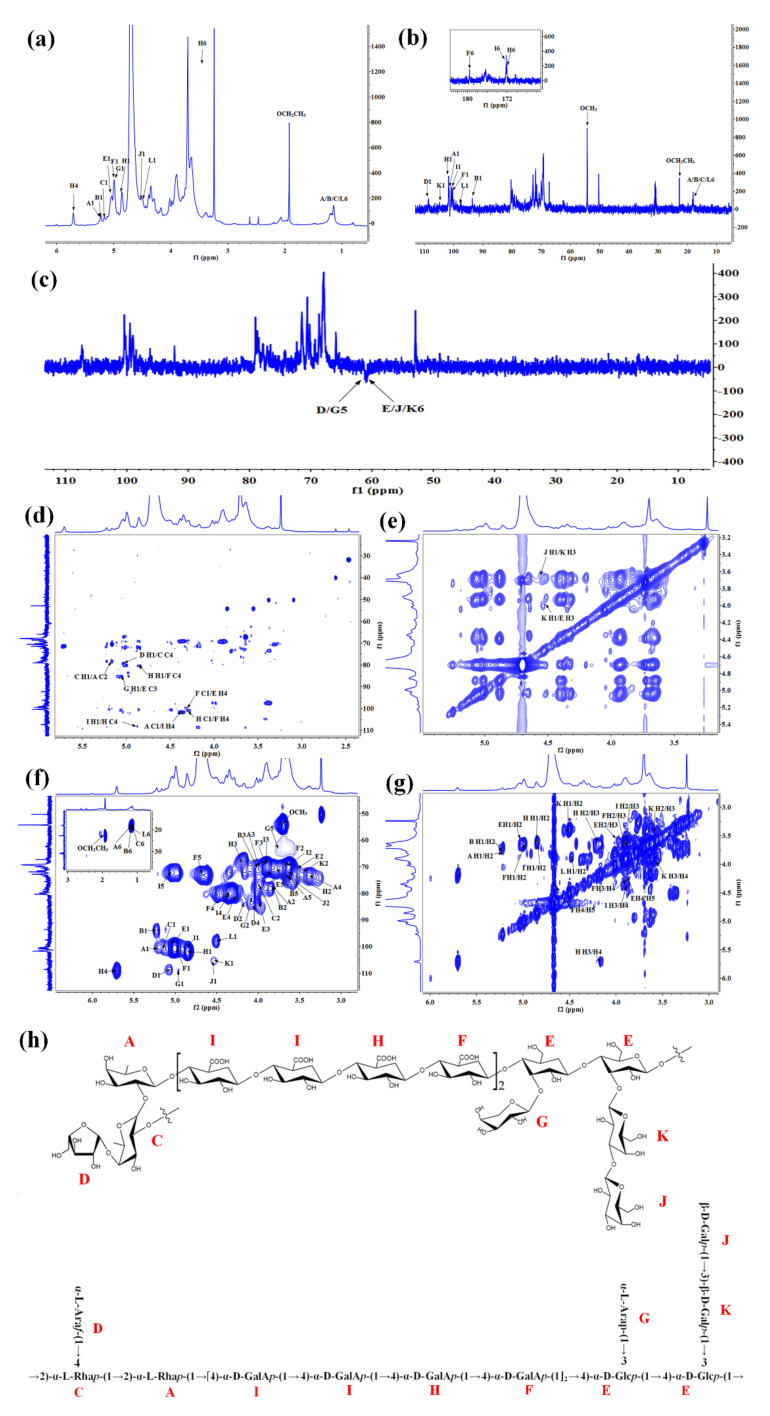
NMR spectra and the suggested structure of RPP-2a. (**a**) ^1^H NMR. (**b**) ^13^C NMR. (**c**) DEPT-135. (**d**) HMBC. (**e**) NOESY. (**f**) HSQC. (**g**) COSY. (**h**) The structure of RPP-2a.

Based on the results of the monosaccharide composition, methylation analysis, and 1D- and 2D-NMR data, the primary chain connection of RPP-2a was found to be →2)-*α*-L-Rha*p*-(1→2)-*α*-L-Rha*p*-(1→[4)-*α*-D-GalA*p*-(1→4)-*α*-D-GalA*p*-(1→4)-*α*-D-GalA*p*-(1→4)-*α*-D-GalA*p*-(1]_2_→4)-*α*-D-Glc*p*-(1→4)-*α*-D-Glc*p*-(1→. The terminal residue *α*-L-Araf-(1→ was located on the backbone of RPP-2a with the O-4 band of rhamnose to form one branch, and the *α*-L-Ara*p*-(1→ and *β*-D-Gal*p*-(1→3)-*β*-D-Gal*p*-(1→ sugar moieties were connected to the O-3 band of glucose to form the other two side chains of RPP-2a. Considering the above analytical results, the suggested structure of RPP-2a is depicted in Figure 6h.

### 2.7. The Effect of RPP-2a on RAW264.7 Macrophages Viability

**Figure 7:**
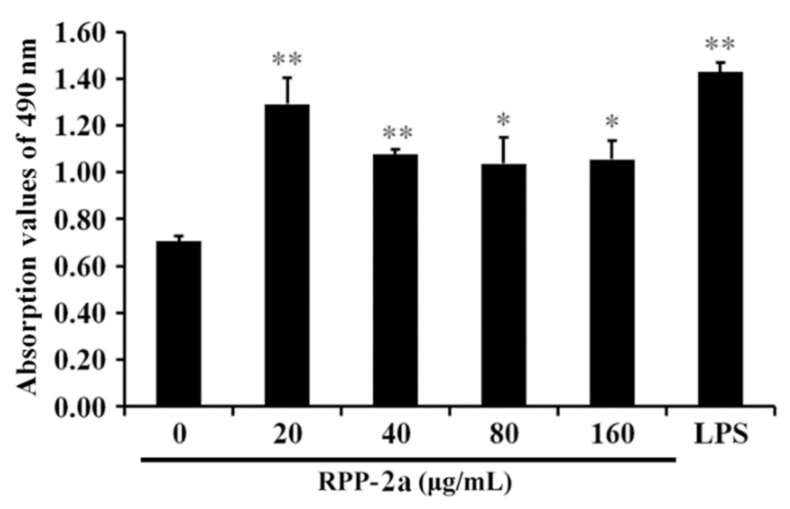
Effect of RPP-2a on the viability of RAW264.7 cells. Data are presented as mean ± SD; * = *p* < 0.05 and ** = *p* < 0.01 vs. control group.

As depicted in Figure 7, RPP-2a had no inhibitory effect on the proliferation of RAW264.7 cells. On the contrary, it significantly promoted the viability of macrophages in the concentration range of 20–160 μg/mL (* indicates *p* < 0.05, and ** indicates *p* < 0.01 vs. the control group). These findings suggested that RPP-2a possesses macrophage activation activity.

### 2.8. Effect of RPP-2a on Nitric Oxide (NO), Tumor Necrosis Factor-α (TNF-α), Interleukin-6 (IL-6), and Interleukin-1β (IL-1β) Production in RAW264.7 Macrophages

**Figure 8:**
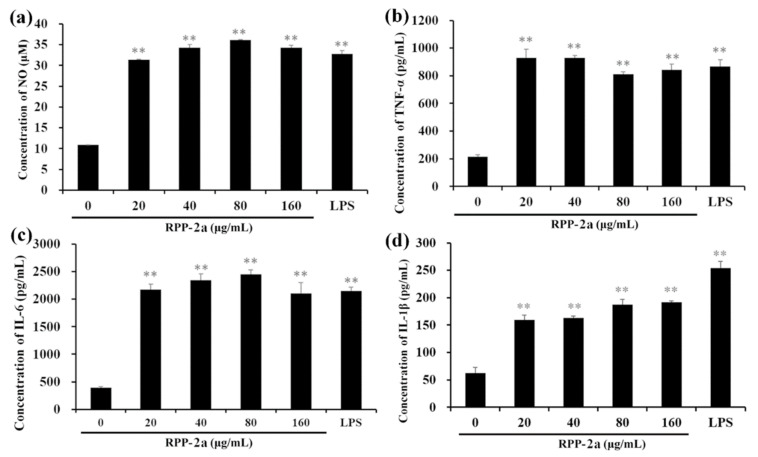
Enhanced effects of RPP-2a on NO and proinflammatory cytokines production. (**a**) NO. (**b**) TNF-α. (**c**) IL-6. (**d**) IL-1β. Data are presented as mean ± SD; ** = *p* < 0.01 vs. control group.

As depicted in Figure 8, after being incubated with RPP-2a, the concentrations of NO, TNF-α, IL-6, and IL-1β in the RAW246.7 cells were significantly increased (** *p* < 0.01 vs. control group) but with different trends. As the RPP-2a concentration increased, the levels of NO and IL-6 showed an increasing trend first, followed by a decreasing trend (Figure 8a,c). However, the opposite phenomenon was observed in the TNF-α secretion (Figure 8b). Moreover, the release of IL-1β was significantly increased in a dose-dependent manner (Figure 8d). The above results further confirmed that RPP-2a induced the activation of macrophages by increasing the production of NO, TNF-α, IL-6, and IL-1β.

### 2.9. Effects of RPP-2a on the Expression of Inducible Nitric Oxide Synthase (iNOS) and Cytokines in RAW264.7 Macrophages

**Figure 9:**
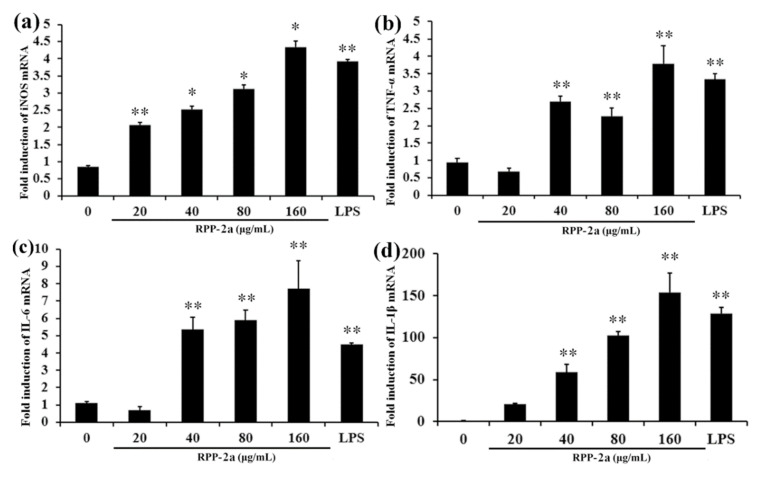
Effects of RPP-2a on the mRNA expression of iNOS and cytokines in RAW264.7 macrophages. The mRNA levels of (**a**) iNOS, (**b**) TNF-α, (**c**) IL-6, and (**d**) IL-1β were measured by qRT-PCR in the RPP-2a treated RAW264.7 cells. Data are presented as mean ± SD, n = 3, * = *p* < 0.05, and ** = *p* < 0.01 vs. control group.

NO production is tightly regulated by iNOS and expressed in the activated macrophages [52]. Thus, it was assessed whether RPP-2a causes the induction of iNOS. As depicted in Figure 9a, RPP-2a promoted mRNA expression of iNOS in RAW264.7 cells in a dose-dependent manner (* *p* < 0.05, ** *p* < 0.01 vs. control group), indicating that RPP-2a enhanced NO production by inducing iNOS expression at the mRNA level. Compared to the vehicle, the mRNA expression of TNF-α (Figure 9b), IL-6 (Figure 9c), and IL-1β (Figure 9d) were significantly stimulated by RPP-2a (with * indicating *p* < 0.05 and ** indicating *p* < 0.01 vs. the control group) in the concentration range of 40–160 μg/mL. These results suggested that the augmentation of RPP-2a on these cytokines occurred at a transcription level, indicating that RPP-2a effectively activated the macrophages.

## 3. Discussion

In this study, an acidic homogeneous polysaccharide RPP-2a was isolated from the pulp of raspberries. The result of monosaccharide composition, methylation analysis, FT-IR, and 1D- and 2D-NMR analyses revealed that the backbone of RPP-2a was composed of →2)-α-L-Rha*p*-(1→2)-α-L-Rha*p*-(1→[4)-α-D-GalA*p*-(1→4)-α-D-GalA*p*-(1→4)-α-D-GalA*p*-(1→4)-α-D-GalA*p*-(1]_2_→4)-α-D-Glc*p*-(1→4)-α-D-Glc*p*-(1→. The terminal residue α-L-Araf-(1→ was located on the backbone of RPP-2a with the O-4 band of rhamnose to form one branch, and the α-L-Ara*p*-(1→ and β-D-Gal*p*-(1→3)-β-D-Gal*p*-(1→ sugar moieties were connected to the O-3 band of glucose to form the other two side chains. The structure of RPP-2a, including the monosaccharide composition, molecular weight, the type of glycosidic linkages, the backbone, and the side chains, was different from that of previously reported polysaccharides obtained from raspberry fruits cultivated in the city Harbin, in China. Taking the monosaccharide composition for example, Ke, et al. have reported that the polysaccharide from *Rubus chingii Hu* was composed of mannose, rhamnose, glucuronic acid, galacturonic acid, glucose, galactose, arabinose and fucose with a molar ratio of 1.31:4.41:1.13:43.20:8.65:9.51:31.17:0.61 [16]. The monosaccharide composition of another raspberry polysaccharide was galacturonic acid, rhamnose, arabinose, xylose, mannose, glucose, and galactose, with a molar ratio of 1.00:0.15:0.65:0.26:0.11:0.10:0.46 [19]. Although the types of monosaccharide in RPP-2a varied between these two raspberry polysaccharides, both were consistent in that galacturonic acid had the highest concentration. Thus, RPP-2a was inferred to be a novel polysaccharide isolated from the raspberry fruits. The variation in the structural features of polysaccharides might be due to the differences in geographical environment and climatic conditions, cultivars, or the extraction and purification procedures [43]. However, the differences in polysaccharides from different raspberry parts could be due to another reason.

The relationship between the structure and immunomodulatory activity is difficult to estimate due to the structural heterogeneity and complex composition of polysaccharides [53]. It is noteworthy that the immunomodulatory activity of a polysaccharide is affected by its conformation, monosaccharide composition, molecular weight, branching degree and functional groups [54]. RPP-2a was composed of rhamnose, arabinose, galactose, glucose, xylose, galacturonic acid, and glucuronic acid, with the highest content being galacturonic acid. Moreover, the monosaccharide composition of polysaccharides influences their biological activities. It is reported that a high percentage of uronic acid might contribute to the immunomodulatory effects of polysaccharides [55]. For instance, Ketha and Gudipati reported that the carboxyl group of uronic acid in mung bean non-starch polysaccharides was involved in the activation of macrophages [56]. Similarly, another in vitro study indicated that galacturonic acid played important roles in the macrophage’s proliferative activity [57]. Thus, RPP-2a might exhibit a strong immunomodulatory activity due to its high galacturonic acid content. Besides monosaccharide composition, the molecular weight might also contribute to the activity of polysaccharides. Lower and higher molecular weight polysaccharides show distinct immune regulation effects in different substances. The lower molecular weight polysaccharides have simpler structural conformation, allowing then to pass through the cell barrier with less hinderance [58]. In contrast, some high molecular weight polysaccharides also have strong immune regulation effects due to the presence of more receptors [59]. The Mw of RPP-2a was 55582 Da, which was not very large, but might be favorable to its immunomodulatory activity. It has been reported that the immunomodulatory activity of pectic polysaccharides is mainly associated with the flexible chain conformations and branching degrees and the removal of branching regions might diminish their immunostimulatory activity [60]. RPP-2a was composed of a branched heteropolysaccharide with ramified chains of *α*-L-Araf-(1→, *α*-L-Ara*p*-(1→, and *β*-D-Gal*p*-(1→3)-*β*-D-Gal*p*-(1→ moieties, contributing to its immunostimulatory activity. Overall, it was speculated that RPP-2a might exhibit a strong immunomodulatory activity.

Macrophages are unique cells in the immune system [61]. After being activated, the macrophages produce NO and release various cytokines, including tumor necrosis factors and interleukins. These cytokines act as the mediators of immune responses to modulate immunity and participate in proinflammatory and anti-inflammatory actions [40]. Moreover, these effectors positively promote the macrophage’s functions [62]. NO is a gaseous molecule that enhances the lysis and phagocytosis of macrophages and is an excellent biomarker for evaluating macrophage activation [63]. TNF-α is the earliest mediator of inflammatory reactions and can promote the activity of macrophages [64]. IL-1β plays a vital role in macrophage activation and acts with TNF-α in inflammation [65]. IL-6 can regulate both the cellular and humoral immunity and participate in phagocytosis, antigen-presenting, and inflammatory regulation [66]. Thus, macrophages are utilized as the potential cell models to evaluate the immunomodulatory activities of bioactive compounds [67]. As a member of the macrophage family, the murine RAW264.7 cells have been widely used for immune activity studies [68]. In this study, RPP-2a significantly promoted the viability of macrophages and increased the levels and mRNA expressions of NO, TNF-α, IL-6, and IL-1β in RAW246.7 cells. these results confirmed that RPP-2a possessed significant macrophage activation activity and exhibited a strong potential to be utilized as a natural immune-enhancing agent. However, the underlying mechanism of the macrophage activation activity of RPP-2a should be investigated in future studies.

## 4. Materials and Methods

### 4.1. Materials

Raspberries (cultivar name: Autumn Britain) were obtained from Qinghai Raspberry Agriculture and Forestry Industrialization Ltd. (Qinghai, China), located in Huangyuan county of Qinghai province (latitude of 101.256, longitude of 36.682 and elevation of 2650 m).

RAW264.7 macrophages were supplied by the Cell Bank of the Chinese Academy of Sciences (Shanghai, China). Dulbecco’s modified eagle medium (DMEM), fetal bovine serum (FBS), penicillin/streptomycin (P/S), dextran standards, and lipopolysaccharide (LPS) were purchased from Sigma-Aldrich Co. (St. Louis, MO, USA). The Cell Counting Kit-8 (CCK-8) and NO detection kit were purchased from Beyotime Biotechnology (Shanghai, China). The enzyme-linked immunosorbent assay (ELISA) kits for TNF-α, IL-6, and IL-1β were purchased from Nanjing Jiancheng Bioengineering Institute (Jiangsu, China). The monosaccharide standards, including rhamnose (Rha), fucose (Fuc), arabinose (Ara), xylose (Xyl), mannose (Man), glucose (Glu), etc., were obtained from The National Institute for Control of Pharmaceutical and Biological Products (Beijing, China). The DEAE Sepharose Fast Flow gel and Sephadex G-200 gel were purchased from Yangzhou BoRui Saccharide Biotech Co. Ltd. (Jiangsu, China). All chemical reagents were of analytical reagent grade.

### 4.2. Preparation and Purification of Polysaccharides from Raspberry Pulp

The crude raspberry pulp polysaccharides (RPPs) were extracted according to the previously reported methods [21]. Briefly, the dried raspberry pulp powder was defatted with petroleum ether (boiling point, 60 °C) at room temperature for 24 h, and then continuously stirred. Finally, it was extracted with 80% ethanol at 60 °C for 2 h to remove the oligosaccharides, colored contaminants, and monosaccharides. The residue was extracted by distilled water at 60 °C for 2 h with ultrasonic assistance. The concentrated portion was subjected to deproteination by the Sevage method three times. Thereafter, a 4-fold volume of 95% ethanol was added to precipitate the polysaccharide at 4 °C overnight. The crude polysaccharides were then collected and freeze-dried for further analyses.

The RPPs were further purified through the DEAE Sepharose Fast Flow chromatography column (7.5 cm × 60 cm) using an NaCl gradient solution (0–0.2 M) as the eluent at a flow rate of 15 mL/min. The eluates were collected automatically, and the polysaccharide content was monitored using the phenol-sulfuric acid method at 490 nm [23]. The fractions were collected, lyophilized, and further purified through a Sephadex G-200 gel permeation column (2.6 cm × 60 cm), and eluted with distilled water at a flow rate of 0.5 mL/min. The fractions were monitored and combined using an HPLC (RID-10A FRC-10A, Shimadzu, Tokyo, Japan) online detection system equipped with a refractive index detector (RI-502, Shodex, Tokyo, Japan). The refined polysaccharide fractions were concentrated, dialyzed, and freeze-dried for further analyses.

### 4.3. Homogeneity and Molecular Weight Determination

The homogeneity and molecular weight of RPP-2a were estimated by a High-Performance Gel Permeation Chromatography (HPGPC) system. The process was performed on an LC-10A HPLC system (Shimadzu, Tokyo, Japan) equipped with a BRT105-104-102 series column (8 mm × 300 mm) maintained at a temperature of 40 °C, and a refractive index detector. The mobile phase was 0.05 M NaCl solution, and the flow rate was 0.6 mL/min. Dextrans with different molecular weights (5000, 11,600, 23,800, 48,600, 80,900, 148,000, 273,000, 409,800, 670,000 Da) were used as the standards.

### 4.4. Infrared Spectrum Analysis

The IR spectrum of RPP-2a was analyzed by a Fourier transform infrared spectrophotometer (FT-IR650, Tianjin Gangdong CO., Hebei, China) based on the potassium bromide disk method within the frequency range of 4000–400 cm^−1^ [36].

### 4.5. Monosaccharide Composition Analysis

The monosaccharide composition of RPP-2a was analyzed by high-performance anion exchange chromatography (HPAEC) [69]. The monosaccharide standards and RPP-2a were converted to their acetylated derivatives according to a previously reported method [70]. A Dionex ICS-5000 system (Thermo Scientific Co., Waltham, MA, USA) equipped with a CarboPac^TM^PA-20 analytical column (3 mm × 150 mm) and a pulsed amperometric detector was employed. Different isocratic schemes with various concentrations of NaOH and sodium acetate (NaOAc) were used to analyze the acidic sugars, with isocratic NaOH (250 mM) for 10 min, followed by NaOAc (500 mM) containing a fixed 50 mM of NaOH for another 30 min. The elution temperature, injection volume, and flow rate were set to 30 ℃, 5 μL, and 0.3 mL/min, respectively.

### 4.6. Methylation and GC-MS Analysis

RPP-2a was methylated, hydrolyzed, reduced, and acetylated, following the previously reported methods [71]. Acetylates were examined using a GC-MS instrument (GCMS-QP-2010, Shimadzu, Tokyo, Japan) equipped with an RXI-5 SIL MS chromatographic column (30 m × 0.25 mm × 0.25 μm). The temperature program was set as follows: the initial column temperature was maintained at 120 ℃, increased to 250 °C at 3 °C/min and maintained for 5 min. The flow rate of H_2_ was 1mL/min and the detector temperature was 250 °C.

### 4.7. 1D and 2D NMR Analysis

RPP-2a (50 mg) was dissolved in D_2_O (0.5 mL), and nuclear magnetic resonance (NMR) spectra were acquired on a 600 MHz spectrometer (Bruker Corp., Fallanden, Switzerland), using a 5 mm probe. For the ^1^H NMR and ^13^C NMR spectra, distortionless enhancement by polarization transfer 135 (Dept135), ^1^H,^1^H chemical-shift correlation spectroscopy (^1^H-^1^H COSY), heteronuclear multiple quantum coherence (HSQC), and heteronuclear multiple-bond correlation (HMBC) experiments were performed.

### 4.8. Cell Culture

RAW264.7 macrophages were cultured in the DMEM medium supplemented with 10% FBS and 1% P/S at 37 ℃ in a 5% CO_2_ atmosphere [72].

### 4.9. Cell Viability

RAW264.7 cells (1 × 10^4^ cells/well) were seeded in a 96-well plate and incubated overnight at 37 ℃. The cells were then treated with various concentrations of RPP-2a (20, 40, 80, 160 μg/mL) or LPS (5 μg/mL) for 24 h, and then 10 μL of CCK-8 was added to each well. After being incubated at 37 ℃ for another 2 h, the absorbance at 490 nm was measured using a spectrophotometer (Thermo Fisher Scientific Oy, Vantaa, Finland).

### 4.10. Determination of NO, TNF-α, IL-6 and IL-1β

The cell seeding and treatment processes were the same as described above in Section 4.9. The NO level in the cell supernatants was assessed using the Griess reagent, and the concentrations of TNF-α, IL-6 and IL-1β in the cell culture medium were determined by the ELISA assay kits according to the manufacturer’s instructions.

### 4.11. Real-Time Quantitative Polymerase Chain Reaction (RT-qPCR)

RAW264.7 cells (2 × 10^6^ cells/well) were seeded into 6-well plates and incubated overnight at 37 °C. The cells were subsequently exposed to a series of concentrations of RPP-2a or LPS for 24 h. The total RNAs were extracted using the RNAsimple Total RNA Kit (TIANGEN, Beijing, China), and then were reverse-transcribed to cDNAs using a FastKing cDNA Dispelling RT SuperMix Kit (TIANGEN, Beijing, China). The RT-qPCR assay was performed on a LightCycler 96 PCR System (Roche, Basel, Switzerland) with SYBR Green (TIANGEN, Beijing, China). The relative expression levels of the target genes were calculated based on 2^−^^△△Ct^. The GADPH gene was used as a housekeeping gene. The primers used in this study are listed in Table 3.

### 4.12. Statistical Analysis

The data were analyzed by a one-way ANOVA, followed by Tukey’s post-hoc test using SPSS version 19.0 software (SPSS, Chicago, IL, USA) and expressed as mean ± standard deviation (SD.). A *p* < 0.05 (*) was considered as a significant difference and *p* < 0.01 (**) was considered as a highly significant difference.

## 5. Conclusions

In this study, a homogeneous acidic polysaccharide, named RPP-2a, was purified from the pulp of raspberries. RPP-2a consisted of rhamnose, arabinose, galactose, glucose, xylose, galacturonic acid, and glucuronic acid, among which the galacturonic acid content was the highest. The Mw, Mn and Mp of RPP-2a were 55582 Da, 38824 Da, and 45471 Da, respectively. The results of FT-IR, GC-MS, 1D, and 2D-NMR analyses suggested that the backbone of RPP-2a was primarily composed of →2)-α-L-Rha*p*-(1→, →2,4)-α-L-Rha*p*-(1→, →4)-α-D-GalA*p*-(1→, and →3,4)-α-D-Glc*p*-(1→, with the side chains of α-L-Ara*f*-(1→, α-L-Ara*p*-(1→, and β-D-Gal*p*-(1→3)-β-D-Gal*p*-(1→ residues linked to the O-4 band of rhamnose and O-3 band of the glucose residues. RPP-2a exhibited significant macrophage activation activity by enhancing NO, TNF-α, IL-6, and IL-1β production, and the expression of iNOS and cytokines at the mRNA level in RAW264.7 cells. These results suggest that RPP-2a can be utilized as a potential natural immune-enhancing agent.

## Figures and Tables

**Table 1:** GC-MS analysis of methylated RPP-2a.

Retention Time (min)	Methylated Sugar	Mass Fragments (*m*/*z*)	Linkages Patterns	Molar Ratios
9.455	2,3,5-Me_3_-Ara*f*	43,71,87,101,117,129,145,161	Ara*f*-(1→	7.61
10.815	2,3,4-Me_3_-Ara*p*	43,71,87,101,117,129,131,161	Ara*p*-(1→	5.69
14.377	3,4-Me_2_-Rha*p*	43,59,87,89,99,115,129,131,189	→2)-Rha*p*-(1→	7.11
16.282	2,3,4,6-Me_4_-Glc*p*	43,71,87,101,117,129,145,161,205	Glc*p*-(1→	1.70
17.459	2,3,4,6-Me_4_-Gal*p*	43,71,87,101,117,129,145,161,205	Gal*p*-(1→	6.22
18.825	3-Me_1_-Rha*p*	43,87,101,117,129,143,159,189	→2,4)-Rha*p*-(1→	5.53
20.511	3,4,6-Me_3_-Gal*p*	43,87,129,161,189	→2-Gal*p*-1→	0.89
21.222	2,3,6-Me_3_-Gal*p*	43,87,99,101,113,117,129,131,161,173,233	→4)-Gal*p*-(1→	44.35
21.409	2,3,6-Me_3_-Glc*p*	43,87,99,101,113,117,129,131,161,173,233	→4)-Glc*p*-(1→	0.66
22.182	2,4,6-Me_3_-Gal*p*	43,87,99,101,117,129,161,173,233	→3)-Gal*p*-(1→	4.33
24.374	2,3,4-Me_3_-Gal*p*	43,87,99,101,117,129,161,189,233	→6)-Gal*p*-(1→	0.79
24.888	2,6-Me_2_-Glc*p*	43,87,97,117,159,185	→3,4)-Glc*p*-(1→	9.81
26.024	3,6-Me_2_-Glc*p*	43,87,99,113,129,173,189,233	→2,4)-Glc*p*-(1→	2.15
27.815	2,3-Me_2_-Gal*p*	43,71,85,87,99,101,117,127,159,161,201	→4,6)-Gal*p*-(1→	2.00
29.536	2,4-Me_2_-Gal*p*	43,87,117,129,159,189,233	→3,6)-Gal*p*-(1→	1.16

**Table 2:** Assignment of ^1^H and ^13^C NMR chemical shift values of RPP-2a.

Sugar	Linkage Type	H1	H2	H3	H4	H5a/H5	H5b/6a	H6b	OCH_3_	OCH_2_CH_3_
		C1	C2	C3	C4	C5	C6			
A	→2)-*α*-L-Rha*p*-(1→	5.22	3.18	3.69	3.4	3.67	1.14			
		101.1	77.86	70.9	73.1	73.2	17.8			
B	→2)-*α*-L-Rha*p*	5.21	3.80	3.96	ns	3.77	1.14			
		93.78	77.86	70.9	ns	71.5	17.8			
C	→2,4)-*α*-L-Rha*p*-(1→	5.17	4.02	3.56	3.86	3.77	1.14			
		99.5	77.86	73.9	78.18	71.5	17.8			
D	*α*-L-Ara*f*-(1→	5.08	4.13	3.87	4.06	3.76	3.64			
		108.45	82.62	77.79	85.22	62.64				
E	→3,4)-*α*-D-Glc*p*-(1→	5.04	3.53	4.01	4.27	3.86	3.92	3.71		
		98.7	70.34	83.3	81.71	73.49	62.26			
F	→4)-*α*-D-GalA*p*-(1→	4.99	3.68	3.92	4.33	4.68				
		100.39	69.8	70.12	79.68	72.92	176.66			
G	*α*-L-Ara*p*-(1→	4.98	4.07	3.96	4.16	3.76	3.64			
		109.23	82.52	78.12	83.6	62.64				
H	→4)-*α*-D-GalA*p*-(1→	4.85	3.64	4.2	5.71			3.71		
		101.88	72.7	67.2	109.16	146.8	172.15			
I	→4)-*α*-D-GalA*p*-(1→	4.84	3.66	3.91	4.36	5.05			3.74	1.96
		100.81	69.54	69.79	80.06	72.96	172.19		54.4	22.2
J	*β*-D-Gal*p*-(1→	4.56	3.63	3.72	3.62	3.67	3.74	3.66		
		105.74	73.12	74.64	69.6	75.9	62.11			
K	→3)-*β*-D-Gal*p*-(1→	4.52	3.47	3.6	3.97	3.64	3.69	3.86		
		104.69	71.97	78.1	70.25	76.4	62.26			
L	→2)-*β*-L-Rha*p*	4.5	4.01	3.96	ns	3.77	1.14			
		97.63	77.86	70.9	ns	71.5	17.8			

The “ns” indicates “no significant difference”.

**Table 3:** PCR primers used in the measurement of mRNA expression.

Target Gene	Primer
GADPH	forward primer: 5′-GGCCTTCCGTGTTCCTACC-3′
	reverse primer: 5′-TGCCTGCTTCACCACCTTC-3′
TNF-α	forward primer: 5′-GCCAGGAGGGAGAACAGAAACT-3′
	reverse primer: 5′-GGCCAGTGAGTGAAAGGGACA-3′
IL-6	forward primer: 5′-GAGGATACCACTCCCAACAGACC-3′
	reverse primer: 5′-AAGTGCATCATCGTTGTTCATACA-3′
IL-1β	forward primer: 5′-GCCACCTTTTGACAGTGATGAG-3′
	reverse primer: 5′-GACAGCCCAGGTCAAAGGTT-3′
iNOS	forward primer: 5′-CCTGTGAGACCTTTGATG-3′
	reverse primer: 5′-CCTATATTGCTGTGGCTC-3′

## Data Availability

Not applicable.
